# The effect of chronic viral hepatitis on prognostic value of inflammatory biomarkers in hepatocellular carcinoma

**DOI:** 10.1002/cam4.3573

**Published:** 2021-07-28

**Authors:** Cortlandt M. Sellers, Johannes Uhlig, Johannes M. Ludwig, Jeffrey S. Pollak, Tamar H. Taddei, Stacey M. Stein, Joseph K. Lim, Hyun S. Kim

**Affiliations:** ^1^ Section of Interventional Radiology Department of Radiology and Biomedical Imaging Yale University School of Medicine New Haven CT USA; ^2^ Department of Radiology Baylor College of Medicine Houston TX USA; ^3^ Department for Diagnostic and Interventional Radiology University Medical Center Goettingen Goettingen Germany; ^4^ Department of Diagnostic and Interventional Radiology and Neuroradiology University Hospital Essen University of Duisburg‐Essen Essen Germany; ^5^ Section of Digestive Diseases Department of Internal Medicine Yale University School of Medicine New Haven CT USA; ^6^ Section of Medical Oncology Department of Internal Medicine Yale University School of Medicine New Haven CT USA; ^7^ Yale Cancer Center Yale University School of Medicine New Haven CT USA

**Keywords:** hepatitis C, carcinoma, hepatocellular, inflammation, neutrophils, lymphocytes

## Abstract

**Background:**

Inflammation and the immune system significantly impact the development, progression, and treatment response of hepatocellular carcinoma (HCC). This retrospective study investigated the neutrophil‐to‐lymphocyte ratio (NLR) as a prognostic biomarker in Western patients with HCC in the setting of chronic viral hepatitis.

**Methods:**

Patients diagnosed with HCC from 2005 to 2016 were selected from a tertiary care institution. NLR was calculated within 30 days prior to treatment and dichotomized at the median. Kaplan–Meier overall survival (OS) curves and Cox hazard proportional models were utilized. Tumor and liver reserve parameters were included in multivariable analyses (MVA).

**Results:**

A total of 581 patients met inclusion criteria (median age 61.0 yr; 78.3% male; 66.3% Caucasian) with median OS = 34.9 mo. 371 patients (63.9%) had viral hepatitis, of which 350 had hepatitis C (94.3%). The low‐NLR group (<median NLR = 2.45) demonstrated higher median OS of 45.6 mo versus the high‐NLR group (median OS 23.9 mo, *p* < 0.0001). Log‐transformed NLR was associated with decreased OS, after multivariable adjustment for confounders (hazard ratio [HR] = 1.34, *p* = 0.0033). Viral hepatitis was identified as an NLR effect modifier: in nonviral hepatitis (*n* = 210), low NLR was associated with higher median OS versus high NLR (56.7 mo vs. 17.6 mo, *p* < 0.0001). This was decreased in viral hepatitis (*n* = 371) (low vs. high NLR: 41.9 mo vs. 35.2 mo, *p* = 0.0109). Further, the interaction term between hepatitis and log‐transformed NLR was significant (*p* = 0.0274) on MVA.

**Conclusions:**

Lower baseline NLR was associated with increased overall survival in HCC. Viral hepatitis serves as an effect modifier of NLR, attenuating its prognostic relevance in this hepatitis C‐predominant population.

## Graphical abstract

Lower baseline NLR was associated with increased overall survival in HCC. Chronic viral hepatitis serves as an effect modifier of NLR, attenuating its prognostic relevance in this hepatitis C‐predominant population.

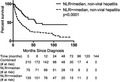

## 1 INTRODUCTION

Hepatocellular carcinoma (HCC) comprises 70%–85% of primary liver cancer; it is the fifth most common cancer and the second leading cause of cancer death worldwide.^1^, ^2^, ^3^ In the United States (U.S.), HCC incidence (6 per 100,000 in 2010)^4^ is increasing, and in East Asia the incidence of HCC is even higher (35.5 per 100,000), likely due to high endemic rates of hepatitis B viral infection (HBV).^5^ It is speculated that up to 50% of HCC cases worldwide are HBV‐related; however, in the United States, over a third of HCC cases are associated with hepatitis C virus (HCV).^6^, ^7^, ^8^ Other common risk factors for HCC include chronic alcohol consumption, nonalcoholic steatohepatitis (NASH), and exposure to aflatoxin.^9^

A growing body of work indicates that inflammation and the immune response play key roles in the development and progression of HCC. Increased levels of tumor‐infiltrating CD8+ T cells have been associated with improved survival in HCC,^10^ while increased numbers of regulatory T cells have been associated with decreased immune response to the tumor, poorer prognosis, and increased risk of metastasis. The neutrophil‐to‐lymphocyte ratio (NLR), platelet‐to‐lymphocyte ratio (PLR), and systemic immune‐inflammatory index (SII) are immune biomarkers that have shown prognostic value in HCC.^11^, ^12^, ^13^ The NLR has also been evaluated as a prognostic marker in multiple other solid organ cancers, including colorectal, renal cell carcinoma, breast, and melanoma.^14^, ^15^, ^16^, ^17^

Despite increasing interest in the NLR and other immune biomarkers in HCC, few have examined their relevance in a Western, HCV‐predominant population. Therefore, the aim of our study was to investigate the relevance of the NLR as a prognostic biomarker in an HCV‐predominant HCC population, with an emphasis on chronic viral hepatitis.

## 2 PATIENTS AND METHODS

### 2.1 Study population

Institutional review board approval was obtained prior to the study, and the protocol was conducted in compliance with the Health Insurance Portability and Accountability Act. We retrospectively analyzed patients from the cancer registry of a single urban academic center diagnosed with HCC based on histopathological or radiological assessment between 2005 and 2016 who received treatments through the hospital system. Informed consent for data usage was obtained from patients at the time of initial treatment. Treatment allocation was determined by a multidisciplinary tumor board and was stratified into systemic therapy, transarterial chemoembolization (TACE), ablation, combined locoregional therapy (combo LRT), resection, and transplantation. Systemic therapy consisted of patients who received chemotherapy with or without radiation. Patients who received both TACE and ablation within a 2‐month period were classified as combo LRT, and patients who received systemic therapy in addition to TACE or ablation were classified as TACE and ablation, respectively. Patients who received resection or transplant following other therapies were classified as resection and transplant, respectively. We excluded individuals under the age of 18, patients with incomplete treatment data, those receiving palliative therapy, and those with a pathologic diagnosis of hepatocholangiocarcinoma.

### 2.2 Data acquisition

Variables reported in the cancer registry included age, gender, ethnicity, American Joint Committee on Cancer (AJCC) staging, treatment status, and vital status. Further treatment and clinical data were acquired through electronic medical record review. Baseline laboratory values and clinical data were used to calculate Child–Pugh and Model for End‐Stage Liver Disease (MELD) scores and Barcelona Clinic Liver Cancer (BCLC) staging at diagnosis. The Charlson Comorbidity Index (CCI)^18^ was used to quantify non‐liver‐related disease burden. Viral hepatitis was defined via a combination of International Classification of Diseases 9th Edition codes for HBV and HCV as well as HBsAg and HCV Ab laboratory data and included patients in all stages of viral hepatitis, including those who were untreated, currently on treatment, or post‐achievement of sustained virologic response. Patients with missing viral hepatitis data were considered as “non‐viral hepatitis.” An HCV subgroup excluded patients with HBV infection only but included patients with HBV/HCV coinfection.

Baseline NLR (neutrophils/lymphocytes), PLR (platelets/lymphocytes), and SII (neutrophils*platelets/lymphocytes) were calculated using lab values drawn within 30 days prior to treatment. Thrombocytopenia was defined as platelets <150,000/μl.

### 2.3 Statistical analysis

Pretreatment immune biomarkers were dichotomized at the median for visualization. For other survival analyses, biomarkers were log‐transformed and evaluated as linear predictors. Categorical variables were compared using the χ^2^ test and continuous variables using the Wilcoxon rank sum test. Survival was estimated using the Kaplan–Meier method and log‐rank test, as well as the Wilcoxon test where survival curves crossed. The Cox proportional hazards method was used to identify predictors of overall survival (OS). Significant predictors in univariate Cox models were tested in multivariable models (MVA) to address confounding. Non‐normally distributed continuous variables were log‐transformed for survival modeling.

An alpha‐level of <0.05 was considered statistically significant. All *p*‐values reported are two‐sided. Calculations were performed and figures created using JMP Pro, R, and GraphPad Prism.

## 3 RESULTS

### 3.1 Demographics

A total of 581 patients met inclusion criteria (Supplemental Figure S1), including 455 men (78.3%), with median age 61.0 years (IQR 56.0–68.0). A total of 385 patients (66.3%) were Caucasian. The etiologies underlying HCC were HCV (*n* = 333, 57.3%), HBV (*n* = 21, 3.6%), combined HBV/HCV (*n* = 17, 2.9%), alcohol (*n* = 65, 11.2%), NASH (*n* = 50, 8.6%), and other (*n* = 95, 16.4%). The majority of patients had Child–Pugh A disease (*n* = 351, 60.4%). TACE was the most frequent treatment utilized (*n* = 155, 26.7%). Seventy patients received systemic therapy (12.0%), of whom 63 (90%) received sorafenib. Additional baseline patient and tumor characteristics are provided in Table 1. Median follow‐up time was 23.4 months, and 333 patients died (57.3%) during the study period.

**TABLE 1:** Baseline characteristics of included patients by neutrophil‐to‐lymphocyte ratio

	Total (*N* = 581)	NLR above median (NLR ≥ 2.45, *N* = 291)	NLR below median (NLR < 2.45, *N* = 290)	*p*‐value
AFP				0.13
Median (IQR)	13.0 ng/ml (5.0–127.0)	11.0 ng/ml (4.0–164.9)	15.0 ng/ml (6.0–107.0)	
ANC (*10^3/μl)				<0.01
Median (IQR)	3.2 (2.2–4.4)	4.1 (3.0–5.5)	2.6 (1.8–3.5)	
ALC (*10^3/μl)				<0.01
Median (IQR)	1.3 (0.8–1.8)	1.0 (0.7–1.4)	1.6 (1.2–2.1)	
Platelets (*10^3/μl)				0.28
Median (IQR)	117.5 (77.0–196.0)	120.0 (72.0–212.3)	115 (78.8–179.3)	
NLR				<0.01
Median (IQR)	2.45 (1.67–3.80)	3.80 (3.12–5.45)	1.67 (1.29–2.04)	
PLR				<0.01
Median (IQR)	95.3 (62.5–142.8)	132.7 (87.1–195.0)	70.6 (49.8–100.1)	
SII				<0.01
Median (IQR)	290.1 (167.0–529.3)	488.5 (288.1–863.4)	190.6 (114.7–290.7)	
Treatment				<0.01
Resection	93 (16.0%)	36 (12.4%)	57 (19.7%)	
Ablation	90 (15.5%)	38 (13.1%)	52 (17.9%)	
Systemic therapy	70 (12.0%)	51 (17.5%)	19 (6.6%)	
Combo LRT	74 (12.7%)	29 (10.0%)	45 (15.5%)	
TACE	155 (26.7%)	81 (27.8%)	74 (25.5%)	
Transplant	99 (17.0%)	56 (19.2%)	43 (14.8%)	
Male gender	455 (78.3%)	236 (81%)	219 (75.5%)	0.10
Age	61.0 (56.0–68.0)	62.0 (57.0–70.0)	60.0 (55.0–68.0)	0.06
Race				<0.01
Black, non‐hispanic	75 (12.9%)	26 (8.9%)	49 (16.9%)	
Hispanic	94 (16.2%)	44 (15.1%)	50 (17.2%)	
Other/unknown	27 (4.7%)	10 (3.4%)	17 (5.9%)	
White, non‐hispanic	385 (66.3%)	211 (72.5%)	174 (60.0%)	
Comorbidities [Charlson Comorbidity Index]	6.0 (5.0–8.0)	7.0 (6.0–8.0)	6.0 (5.0–7.0)	<0.01
Viral hepatitis				<0.01
Viral hepatitis	371 (63.9%)	156 (53.6%)	215 (74.1%)	
None	210 (36.1%)	135 (46.4%)	75 (25.9%)	
Etiology of HCC				<0.01
HCV	333 (57.3%)	135 (46.4%)	198 (68.3%)	
HBV	21 (3.6%)	8 (2.8%)	13 (4.5%)	
HBV/HCV	17 (2.9%)	13 (4.5%)	4 (1.4%)	
Ethanol	65 (11.2%)	43 (14.8%)	22 (7.6%)	
NASH	50 (8.6%)	29 (10.0%)	21 (7.2%)	
Other	95 (16.4%)	63 (21.7%)	32 (11.0%)	
Child–pugh score				<0.01
A	351 (60.4%)	149 (51.2%)	202 (69.7%)	
B	163 (28.1%)	100 (34.4%)	63 (21.7%)	
C	57 (9.8%)	38 (13.1%)	19 (6.6%)	
MELD score	9.0 (7.0–13.0)	10.0 (7.0–14.0)	8.5 (7.0–11.0)	<0.01
BCLC score				<0.01
0 or A	132 (22.7%)	51 (17.5%)	81 (27.9%)	
B	84 (14.5%)	40 (13.7%)	44 (15.2%)	
C	268 (46.1%)	143 (49.1%)	125 (43.1%)	
D	67 (11.5%)	45 (15.5%)	22 (7.6%)	
AJCC stage				<0.01
1	266 (45.8%)	124 (42.6%)	142 (49.0%)	
2	162 (27.9%)	73 (25.1%)	89 (30.7%)	
3	80 (13.8%)	50 (17.2%)	30 (10.3%)	
4	43 (7.4%)	28 (9.6%)	15 (5.2%)	
Tumor size				<0.01
Median (IQR)	2.9 (2.0–4.8)	3.2 (2.2–5.8)	2.8 (2.0–4.2)	
Multifocal HCC				0.08
Yes	233 (40.1%)	127 (43.6%)	106 (36.6%)	
No	347 (59.7%)	163 (56.0%)	184 (63.4%)	
HCC location				0.04
Unilobar	411 (70.7%)	192 (66.0%)	219 (75.5%)	
Bilobar	162 (27.9%)	91 (31.3%)	71 (24.5%)	
Vascular invasion	95 (16.4%)	60 (20.6%)	35 (12.1%)	0.02
Extrahepatic metastases	58 (10.0%)	42 (14.4%)	16 (5.5%)	<0.01

Abbreviations: AFP, alpha‐fetoprotein; AJCC, American Joint Committee on Cancer staging system; ALC, absolute lymphocyte count; ANC, absolute neutrophil count; BCLC, Barcelona Clinic Liver Cancer staging; HBV, hepatitis B virus; HCC, hepatocellular carcinoma; HCV, hepatitis C virus; IQR, interquartile range; LRT, locoregional therapy; MELD, Model of End‐stage Liver Disease; NASH, nonalcoholic steatohepatitis; NLR, neutrophil‐to‐lymphocyte ratio; PLR, platelet‐to‐lymphocyte ratio; SII, systemic immune‐inflammatory index; TACE, transarterial chemoembolization.

### 3.2 Overall survival of the cohort

The median overall survival time was 34.9 months, with 1‐, 3‐, and 5‐year survival rates of 75.2%, 48.7%, and 34.9%. Transplant patients had the highest survival rates, with median OS not reached and 5‐year OS of 86.2%, followed by resection (median OS 51.1 mo), ablation (36.5 mo), combo LRT (27.9 mo), TACE (22.2 mo), and systemic therapy (5.1 mo; overall *p* < 0.0001) (Supplemental Figure S2). Univariate predictors associated with decreased survival (*p* < 0.05) included older age, increased comorbidities, nonviral hepatitis, higher Child–Pugh score, advanced BCLC stage, advanced AJCC stage, increased tumor size, multifocal tumors, bilobar tumor burden, vascular invasion, and extrahepatic metastases as well as systemic therapy and increased alpha‐fetoprotein (AFP). On multivariable analysis, decreased survival was associated with Child–Pugh B disease, BCLC stage D, AJCC stage IV, bilobar tumors, nonviral hepatitis, systemic therapy, and AFP (*p* < 0.05). (See Table 2).

**TABLE 2:** Prognostic factors in hepatocellular carcinoma on multivariable analysis

Predictor	HR	Lower.95.CI	Upper.95.CI	*p*‐value
Log (NLR)	1.34	1.10	1.63	<0.01
Log (AFP)	1.08	1.02	1.14	<0.01
Viral hepatitis	0.67	0.50	0.90	<0.01
Child–pugh class				
A (reference)				
B	2.18	1.55	3.07	<0.01
C	0.37	0.15	0.94	<0.05
BCLC stage				
0 or A (reference)				
B	1.57	0.95	2.57	0.08
C	1.37	0.91	2.07	0.13
D	5.67	2.54	12.64	<0.01
AJCC stage				
1 (reference)				
2	1.29	0.91	1.82	0.15
3	1.82	1.18	2.82	<0.01
4	2.19	1.25	3.82	<0.01
Tumor location				
Unilobar (reference)				
Bilobar	1.78	1.29	2.46	<0.01
Treatment allocation				
Resection (reference)				
Transplant	0.14	0.07	0.28	<0.01
Ablation	1.26	0.66	2.42	0.49
Combo LRT	2.11	1.12	3.97	0.02
TACE	1.54	0.88	2.69	0.13
Systemic therapy	4.49	2.31	8.74	<0.01

Abbreviations: AFP, alpha‐fetoprotein; AJCC, American Joint Committee on Cancer staging system; CI, confidence interval; HCC, hepatocellular carcinoma; HR, hazard ratio; NLR, neutrophil‐to‐lymphocyte ratio.

### 3.3 Immune biomarkers

**FIGURE 1:**
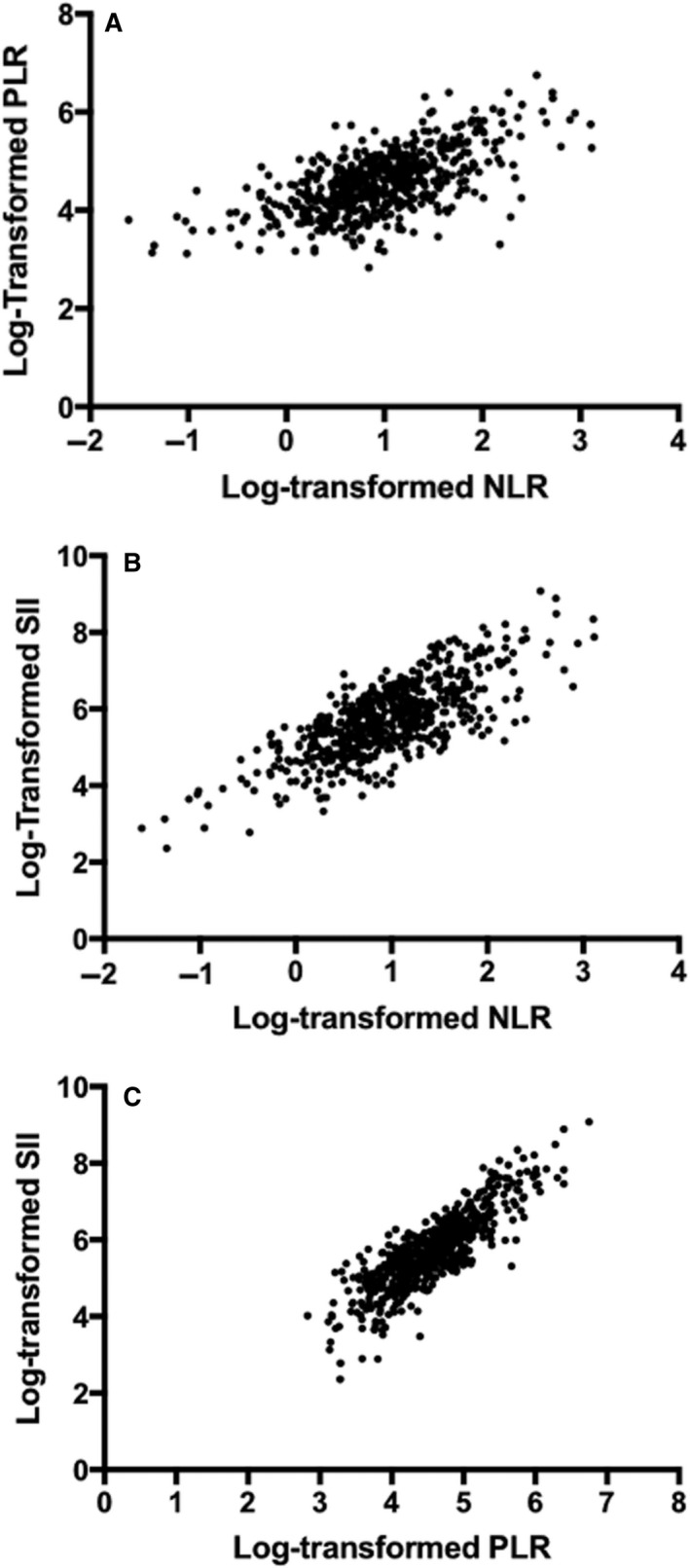
Correlation between log‐transformed inflammatory biomarkers. (A) Platelet‐to‐lymphocyte ratio by neutrophil‐to‐lymphocyte ratio (Spearman ρ = 0.62). (B) Systemic immune‐inflammatory index by neutrophil‐to‐lymphocyte ratio (Spearman ρ = 0.74) (C) Systemic immune‐inflammatory index by platelet‐to‐lymphocyte ratio (Spearman ρ = 0.85)

Median absolute neutrophil count (ANC) for the cohort was 3.2*10^3/μl, with median absolute lymphocyte count (ALC) of 1.3*10^3/μl and median platelet count of 117.5*10^3/μl. The median NLR was 2.45, median PLR was 95.28, and median SII was 290.05. As depicted in Figure 1a–c, there was good to very strong correlation between the inflammatory markers. In separate multivariable Cox models adjusting for potential confounders, NLR, PLR, and SII each emerged as significant survival predictors. However, when including all three biomarkers in one multivariable analysis, only NLR remained significant. Therefore, the remainder of our analyses were conducted using NLR as the sole biomarker.

Patients in the low‐NLR group had higher rates of African American race (16.9%) or Hispanic ethnicity (17.2%) than patients in the high‐NLR group (8.9% and 15.1%, respectively, *p* = 0.0048). Decreased NLR was associated with lower comorbidity indices, higher rates of viral hepatitis; lower MELD and Child–Pugh scores; lower BCLC and AJCC staging, lower tumor size, less vascular invasion, higher rates of unilobar tumors, and fewer extrahepatic metastases (*p* < 0.05). The low‐NLR cohort had lower rates of alcoholic hepatitis (7.6%) or NASH (7.2%) versus patients in the high‐NLR group (14.8% and 10.0%, respectively, overall *p* < 0.0001) and also had higher rates of ablation, combo LRT, and resection and lower rates of systemic therapy, TACE, or transplant as compared to the high‐NLR group.

**FIGURE 2:**
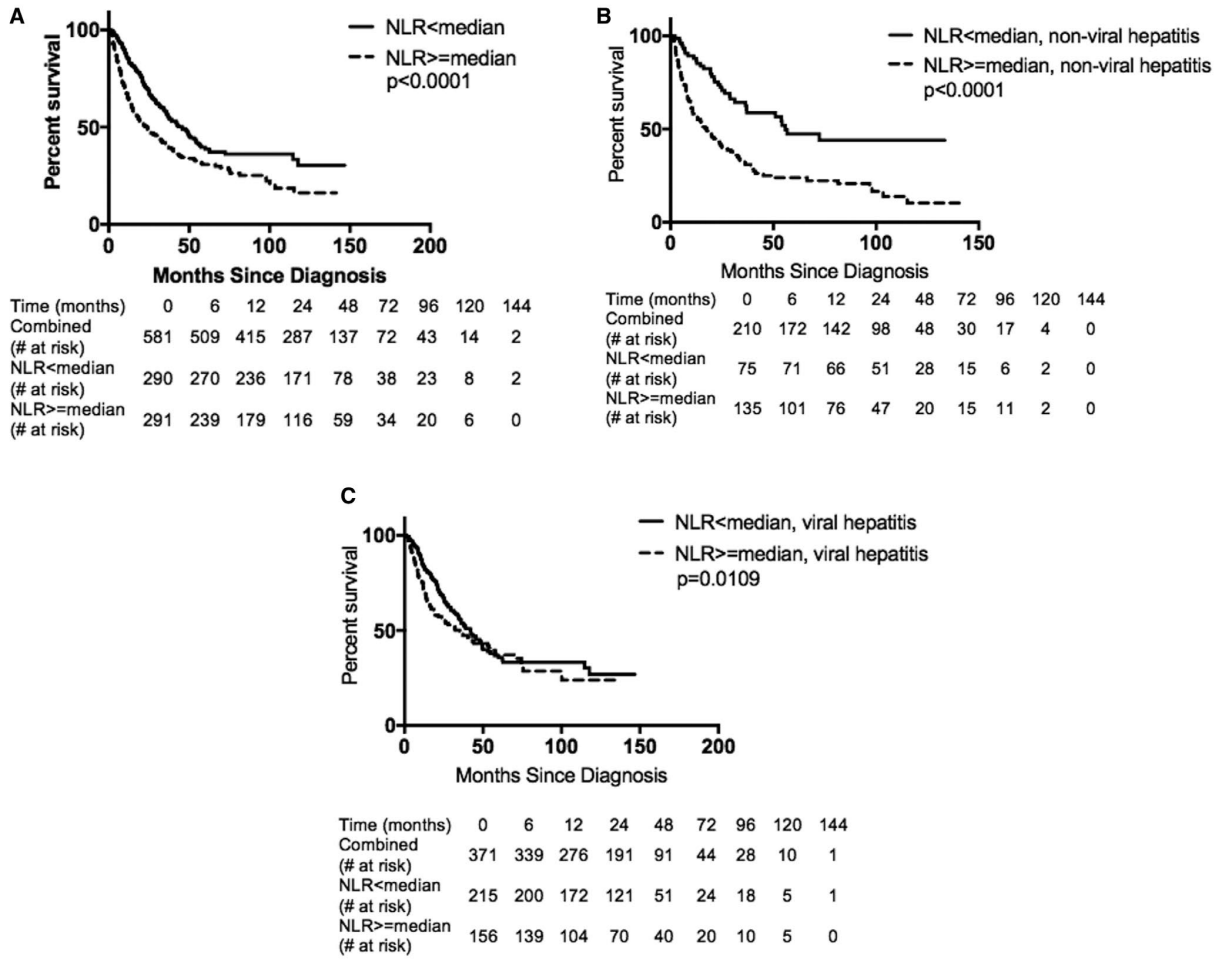
Kaplan–Meier curves of survival from diagnosis of HCC by NLR group. (A) Entire cohort (log‐rank test, *p* < 0.0001). (B) Nonviral hepatitis (log‐rank test, *p* < 0.0001). (C) Viral hepatitis (Wilcoxon test, *p* = 0.0109)

Log‐transformed NLR was associated with decreased OS after multivariable adjustment for confounders (HR = 1.34, 95% CI: 1.10–1.63, *p* = 0.0033). The low‐NLR group (<median NLR = 2.455) demonstrated higher median OS of 45.6 mo versus the high‐NLR group (median OS 23.9 mo, *p* < 0.0001, see Figure 2a). The impact of NLR was then tested across treatment subgroups. Notably, the effect of NLR was strongest in systemic therapy (low vs. high NLR: median OS 12.3 mo vs. 4.1 mo, *p* = 0.0008); TACE (31.5 mo vs. 16.4 mo, *p* = 0.0018); and resection (62.4 mo vs. 42.8 mo, *p* = 0.0357). While a similar trend was seen in the low‐NLR group for ablation, combo LRT, and transplant, these did not reach statistical significance.

### 3.4 Viral hepatitis and neutrophil‐to‐lymphocyte ratio

Sixty‐four percent of patients had viral hepatitis (*n* = 371), of which 333 (89.8%) were HCV, 21 (5.6%) were HBV, and 17 (4.6%) were HBV/HCV coinfection. A total of 137 HCV patients (39.3%) received direct‐acting antiviral therapy (DAA), with 31 (22.3%) having started DAA therapy prior to HCC diagnosis. The viral hepatitis group had higher survival from diagnosis of HCC (median OS 38.9 mo) versus nonviral hepatitis patients (27.7 mo, *p* = 0.0286).

Patients with viral hepatitis were more likely to be younger, male, and African American (*p* < 0.05) and had lower comorbidity indices, lower AJCC staging, higher median AFP, and decreased median tumor size (*p* < 0.05) (Table 1). Patients with viral hepatitis had lower rates of systemic therapy, resection, and TACE and higher rates of ablation, combo LRT, and transplant (*p* < 0.01). While patients with viral hepatitis had higher rates of BCLC stage 0 or A disease and lower rates of BCLC stage B disease compared to patients with nonviral hepatitis, rates of BCLC stage C and D disease were similar between the two groups. Patients with and without viral hepatitis had similar distributions of Child–Pugh score, MELD score, unilobar versus bilobar tumor burden, multifocal tumors, vascular invasion, and extrahepatic metastases.

HCC patients with viral hepatitis had lower ANC versus patients with nonviral hepatitis (median 3.0*10^3/μl vs. 3.7*10^3/μl, *p* < 0.0001) and higher platelet counts (median 110*10^3/μl vs. 149*10^3/μl, *p* < 0.0001). Median ALC was similar between the two groups (1.3*10^3/μl vs. 1.3*10^3/μl, *p* = 0.0502). Patients with viral hepatitis had higher rates of thrombocytopenia (68.5%) versus nonviral hepatitis (50.0%, *p* < 0.0001). The median NLR was higher in nonviral hepatitis patients (3.0) versus viral hepatitis patients (2.2, *p* < 0.0001), as was the median PLR (123.3 vs. 78.3, *p* < 0.0001) and the median SII (445.9 vs. 237.8, *p* < 0.0001).

On multivariable analyses, viral hepatitis was identified as an effect modifier for NLR: the interaction term between hepatitis status and log‐transformed NLR was significant on MVA (*p* = 0.0274). In nonviral hepatitis patients, low NLR was associated with increased survival (Figure 2b; median OS 56.7 vs. 17.6 mo, *p* < 0.0001). However, in the presence of viral hepatitis, this prognostic relevance was attenuated (Figure 2c; low vs. high NLR: median OS 41.9 vs. 35.2 mo, *p* = 0.0109). Similar results were found for the HCV subgroup; however, the interaction term between HCV status and the log‐transformed NLR was nonsignificant (*p* = 0.0537), likely due to lower power in the HCV subgroup due to smaller sample size.

## 4 DISCUSSION

### 4.1 NLR in HCC

An estimated 70%–85% of primary liver cancers are due to HCC, and up to 60% of HCC globally arise from HBV or HCV infection.^1^, ^6^, ^19^ Increased NLR has been described as a biomarker associated with decreased overall survival in multiple solid tumors, including HCC.^11^, ^14^, ^15^, ^16^, ^17^ The NLR has been examined in HCC following transplant,^20^ resection,^21^ radiofrequency ablation,^22^ TACE,^23^, ^24^ radioembolization,^25^ and sorafenib treatment.^26^ However, while the utility of the NLR has been reported in HBV‐related HCC, few have analyzed the effect of the NLR in an HCC population with high rates of HCV infection.^11^, ^27^ Moreover, the majority of studies have been conducted in European or Asian populations, and their results might not be generalizable to a more diverse U.S. population; Sullivan et al. utilized regression models to analyze the predictive value of the NLR in a Western (United States) population of HCC and did not find the NLR to be a significant predictor of survival.^28^

Upon assessing the prognostic impact of NLR, PLR, and SII in HCC, NLR emerged as the single most relevant biomarker and was independently associated with decreased overall survival. This effect carried through in our subgroup analyses to patients treated with systemic therapy, TACE, and resection. A trend toward decreased survival with increasing NLR was also seen in the combined LRT, ablation and transplant subgroups. The NLR appeared to have the strongest impact in patients receiving systemic therapy.

### 4.2 NLR and viral hepatitis

Chronic viral hepatitis served as an effect modifier for the prognostic value of the NLR. While the hepatitis and nonviral hepatitis cohorts differed by BCLC stage and mean tumor size, the interaction term between log‐transformed NLR and hepatitis status was significant on MVA adjusting for those factors and others. The NLR was less impactful in patients with viral hepatitis than patients without viral hepatitis. This may in part be due to the effects of chronic inflammation on the immune response to HCC. Our findings further demonstrated that HCC patients with chronic viral hepatitis had lower neutrophil and platelet counts and lower median biomarkers (NLR, PLR, and SII), as well as higher rates of thrombocytopenia. Liver disease burden as assessed by Child–Pugh and MELD scores was similar between the two groups, suggesting that the increased thrombocytopenia of the viral hepatitis cohort may be related to factors beyond cirrhosis.

While decreased NLR in our study was associated with fewer comorbidities (possibly due to decreased systemic disease burden and decreased systemic inflammation), less advanced liver disease, less advanced cancer staging, and smaller tumors, log‐transformed NLR remained an independent predictor of overall survival on multivariable analysis. This suggests that the mechanism of action of the NLR may not be through severity of liver disease or stage of HCC but through other mechanisms.

### 4.3 Immune mechanisms

The interplay between cancer formation and the immune system is complex. For example, neutrophils have been found to have both pro‐tumor and antitumor properties.^29^ Chronic inflammation, such as that which occurs in the setting of chronic HBV and HCV infection, has been associated with increased susceptibility for the development of cancers.^30^ Bolte et al. found decreased intrahepatic mucosal‐associated invariant T cells in patients with chronic HCV as compared to controls.^31^ These chronic infections affect the immune system by creating a constant cycle of inflammation, necrosis, and regeneration and through activation of CD8+ T and natural killer cells as well as the production of reactive oxygen species and resultant DNA damage.^30^, ^32^, ^33^

Although our study focused solely on circulating markers of inflammation, it is plausible that the NLR may be associated with changes in the tumor microenvironment. There is no current consensus about how the NLR affects the immunologic composition of HCC. A meta‐analysis by Zhang and colleagues reported that both increased peripheral blood T‐regs and increased intratumoral T‐regs were associated with poorer survival in HCC.^34^ Conversely, although Sun concluded that high levels of intratumoral regulatory T cells (T‐regs) were a negative prognostic factor, high levels of peritumoral T‐regs were not.^35^ Further work is required to establish the precise relationship between systemic inflammatory markers and local inflammation in HCC and its microenvironment.

Of the treatments examined in subgroup analyses, the NLR had the strongest effect in systemic chemotherapy patients, with survival in the low‐NLR group being almost three times as high as survival in the high‐NLR group. The majority of these patients (90%) received sorafenib, which is a tyrosine kinase inhibitor (TKI) that inhibits tumor angiogenesis and proliferation while increasing apoptosis^36^, ^37^. TKIs have also been demonstrated to have immunomodulatory effects on non‐tumoral cells, such as a sharp decline in pro‐inflammatory cytokines^38^ and decreased T‐regs^39^, ^40^, possibly secondary to inhibited proliferation and induced apoptosis. Patients with a low baseline NLR may have higher baseline populations of T‐regs than patients with a high NLR, which may affect their response to sorafenib.

Future studies examining the NLR could focus not only on sorafenib, but also on the several other oral systemic therapies which have been recently approved. In addition, when studying the efficacies of systemic therapies, it may be prudent to stratify analyses by viral or nonviral HCC, particularly since subgroup analyses of the SHARP trial demonstrated significant benefit in HCV patients,^41^ who would likely have a lower NLR.

### 4.4 Limitations

Our study included a diverse, well‐characterized cohort regarding age, gender, race, and ethnicity as well as diverse treatment modalities. Our sample size is a limiting factor in subgroup analyses; however, our HCC cohort is among the largest studies in which NLR has been examined. While this study relied on archived records, we limited the NLR values to within 30 days of HCC treatment in order to improve standardization. Our study had low rates of DAA initiation or completion prior to HCC diagnosis. HCV‐predominant HCC cohorts with higher rates of DAA completion prior to HCC diagnosis may demonstrate varying impact of the NLR. This study was conducted among a U.S. population, and results may differ in other parts of the world.

## 5 CONCLUSIONS

Lower baseline neutrophil‐to‐lymphocyte ratios are associated with decreased survival in Western patients with HCC. The neutrophil‐to‐lymphocyte ratio is a superior prognostic marker to the platelet‐to‐lymphocyte ratio and systemic immune‐inflammatory index. The effect of the NLR is decreased in patients with a history of viral hepatitis in this cohort of hepatitis C‐predominant HCC. Further studies are warranted to investigate the impact of chronic viral hepatitis on tumor inflammation, immune response, and survival in HCC, particularly at a local or tumor level.

## CONFLICT OF INTEREST

The author has no conflicts of interest to report for this article. The author certifies that they have no affiliations with or involvement in any organization or entity with any financial interest in the subject matter or materials discussed in this manuscript. HSK has served on Advisory Boards for Boston Scientific, Sirtex, Genentech, Eisai, Amgen, and Bayer, and was supported of research device support from Galil and database from Flatiron Health. JU received speaker fees from Bayer Vital GmbH on a subject not related to the study at hand.

## AUTHORS' CONTRIBUTIONS

CMS, JU, JML, and HSK contributed to conceptualization, formal analysis, methodology, and reviewing and editing the manuscript. CMS was responsible for data curation, funding acquisition, and writing the draft manuscript. JKL, JP, TT, and SS contributed to reviewing and editing the manuscript. HSK supervised this project. All authors read and approved the final manuscript.

## Supporting information

Fig S1

Fig S2

## Data Availability

The data sets generated and analyzed during the current study are not publicly available due to institutional privacy requirements but may be available from the corresponding author on reasonable request.
