# Supplementary figures and images for: The effect of chronic viral hepatitis on prognostic value of inflammatory biomarkers in hepatocellular carcinoma
Source: Cancer Med. 2021 Jul 28;10(16):5395–404. doi: 10.1002/cam4.3573 (PMC8366096; doi:10.1002/cam4.3573)

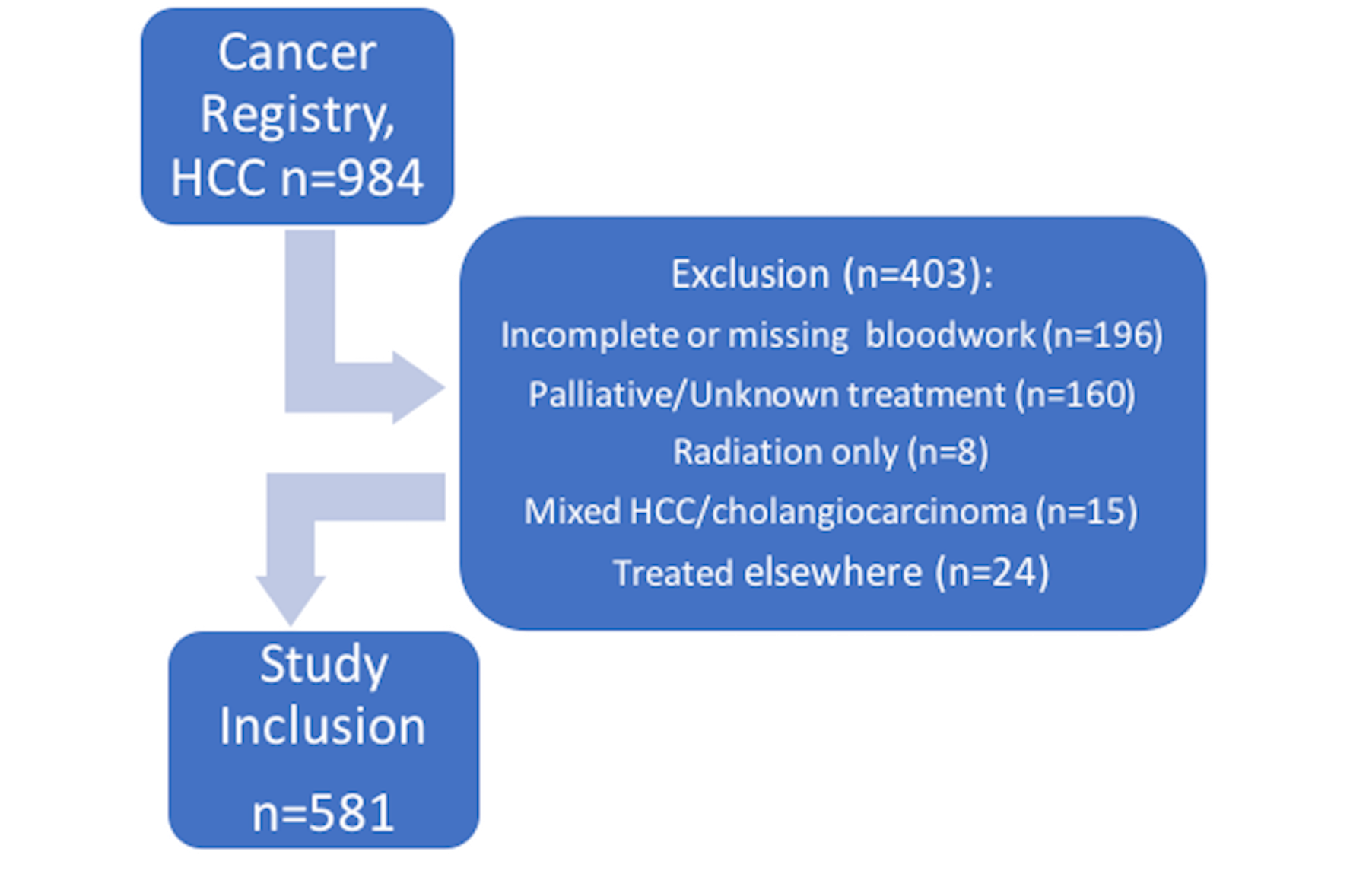

Supplement: Supplementary file 1 — Fig S1 [file CAM4-10-5395-s001.tif]

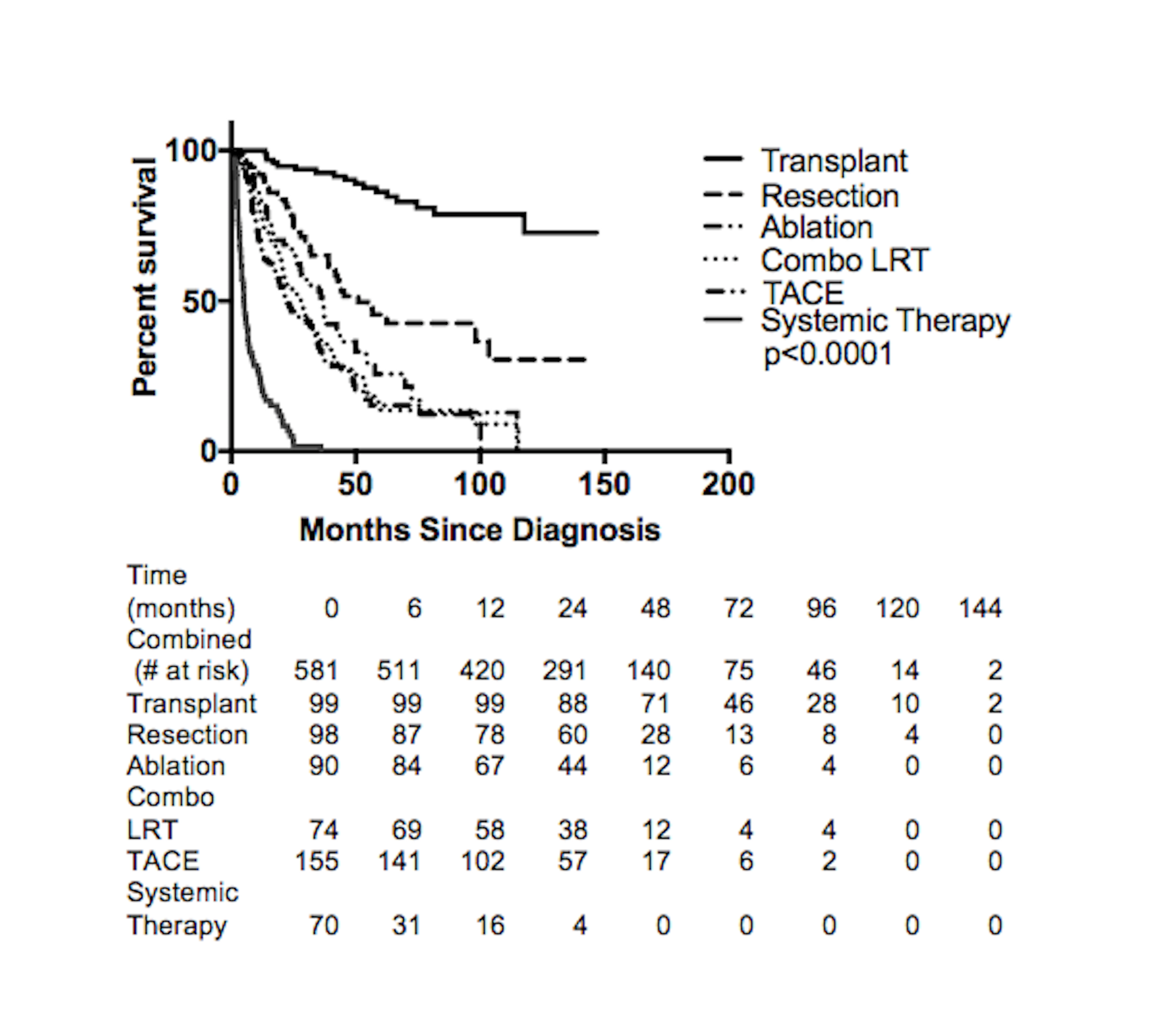

Supplement: Supplementary file 2 — Fig S2 [file CAM4-10-5395-s002.tif]
